# 

**Published:** 1879-07

**Authors:** 


THE BUFFALO

### MEDICAL AND SURGICAL

### JOURNAL.

VOL. XVIII. JULY, 1879. NO. 12.

